# A Well-Controlled Nucleus Pulposus Tissue Culture System with Injection Port for Evaluating Regenerative Therapies

**DOI:** 10.1007/s10439-015-1428-y

**Published:** 2015-08-21

**Authors:** Irene T. M. Arkesteijn, Vivian H. M. Mouser, Fackson Mwale, Bart G. M. van Dijk, Keita Ito

**Affiliations:** Orthopaedic Biomechanics, Department of Biomedical Engineering, Eindhoven University of Technology, PO Box 513, 5600 MB Eindhoven, The Netherlands; Department of Orthopaedics, University Medical Center Utrecht, Utrecht, The Netherlands; Division of Orthopaedic Surgery, Lady Davis Institute for Medical Research, McGill University, Montreal, Canada

**Keywords:** Intervertebral disc, Regeneration, *In vitro*, Link-N, TNF-α

## Abstract

*In vitro* evaluation of nucleus pulposus (NP) tissue regeneration would be useful, but current systems for NP culture are not ideal for injections. The aim of this study was to develop a long-term culture system for NP tissue that allows injections of regenerative agents. Bovine caudal NPs were harvested and placed in the newly designed culture system. After equilibration of the tissue to 0.3 MPa the volume was fixed and the tissue was cultured for 28 days. The cell viability and extracellular matrix composition remained unchanged during the culture period and gene expression profiles were similar to those obtained in earlier studies. Furthermore, to test the responsiveness of bovine caudal NPs in the system, samples were cultured for 4 days and injected twice (day 1 and 3) with (1) PBS, (2) Link-N, for regeneration, and (3) TNF-α, for degeneration. It was shown that TNF-α increased *COX2* gene expression, whereas no effect of Link-N was detected. In conclusion, the newly designed system allows long-term culture of NP tissue, wherein tissue reactions to injected stimulants can be observed.

## Introduction

Intervertebral discs support high magnitude loads and allow multi-directional flexibility in the spine, due to an ingenious interplay of the tissues within the disc. The highly hydrated nucleus pulposus (NP) evenly distributes compressive stresses to the organized annulus fibrosus (AF), which balances these with internal tensile stresses.^31^ The human NP starts to degenerate early in adulthood, resulting in a non-uniform stress distribution, subsequent disorganization and disruption of the AF, and potentially pain.^36^ It has been suggested that early stage NP regeneration could halt this degenerative cascade.^12^ Biological therapies for NP regeneration aim at restoring the water and proteoglycan content of the tissue. For this purpose exogenous cells, to produce extracellular matrix, and molecular agents or gene therapy, to increase the matrix production by resident cells, can be introduced into the tissue.^12^ However promising, these methods still face challenges and require more extensive testing.

Currently, the most widely used model to test regenerative therapies is the *in vivo* animal model. Its main advantage is the presence of all natural interactions of the organism. However in most test cases, rodents or other small animals are used in which degeneration is artificially induced. First of all, such small animals have a mature nuclear cell type, which is different than that of humans.^1^ Second, the artificially induced degeneration is different from the human pathophysiological degenerative cascade. Thus, translation of results obtained in studies with artificial degeneration to naturally degenerated human NPs is difficult.^2^ Testing regenerative therapies on *in vitro* cultured cells is another standard method. However, cell metabolism and phenotype outside of the native matrix and environment change.^9^ Hence, tissue culture models are of great interest.

Although both organ and NP tissue culture models are available, long-term organ cultures are still under development.^4^,^10^ As the evaluation of regenerative therapies often requires long-term studies, we focused on optimizing currently available NP culture systems. The advantage of the NP culture system is the presence of cell–matrix interactions in a controlled culture environment. In standard culture conditions NP tissue does not thrive in the long-term, because it swells tremendously. Previously designed culture systems for NP explants have demonstrated that the tissue matrix can be maintained when it is physically constrained.^11^,^30^ Furthermore, when explants were cultured at physiological oxygen, pH and glucose levels for 42 days, matrix protein gene expression was nearly preserved.^30^ However, our existing system has two major drawbacks: the swelling pressure cannot be standardized, limiting the reproducibility, and repeated administration of injections is not feasible, as these damage the culture system.

Therefore, the aim of this study is to design and develop a novel NP culture system, which allows prevention of tissue swelling in a controlled manner and repeated injections of regenerative agents. It was hypothesized that bovine NP tissue, when first equilibrated to a physiological pressure of 0.3 MPa^33^ in this new system, is responsive to injected stimuli. In a first experiment, NP tissue was cultured long-term to test the maintenance of the extracellular matrix and gene expression in the system. Then, the response to injections of regenerative peptide Link-N or inflammatory cytokine TNF-α was evaluated in a short-term experiment.

## Materials and Methods

### Culture System Design

**Figure 1:**
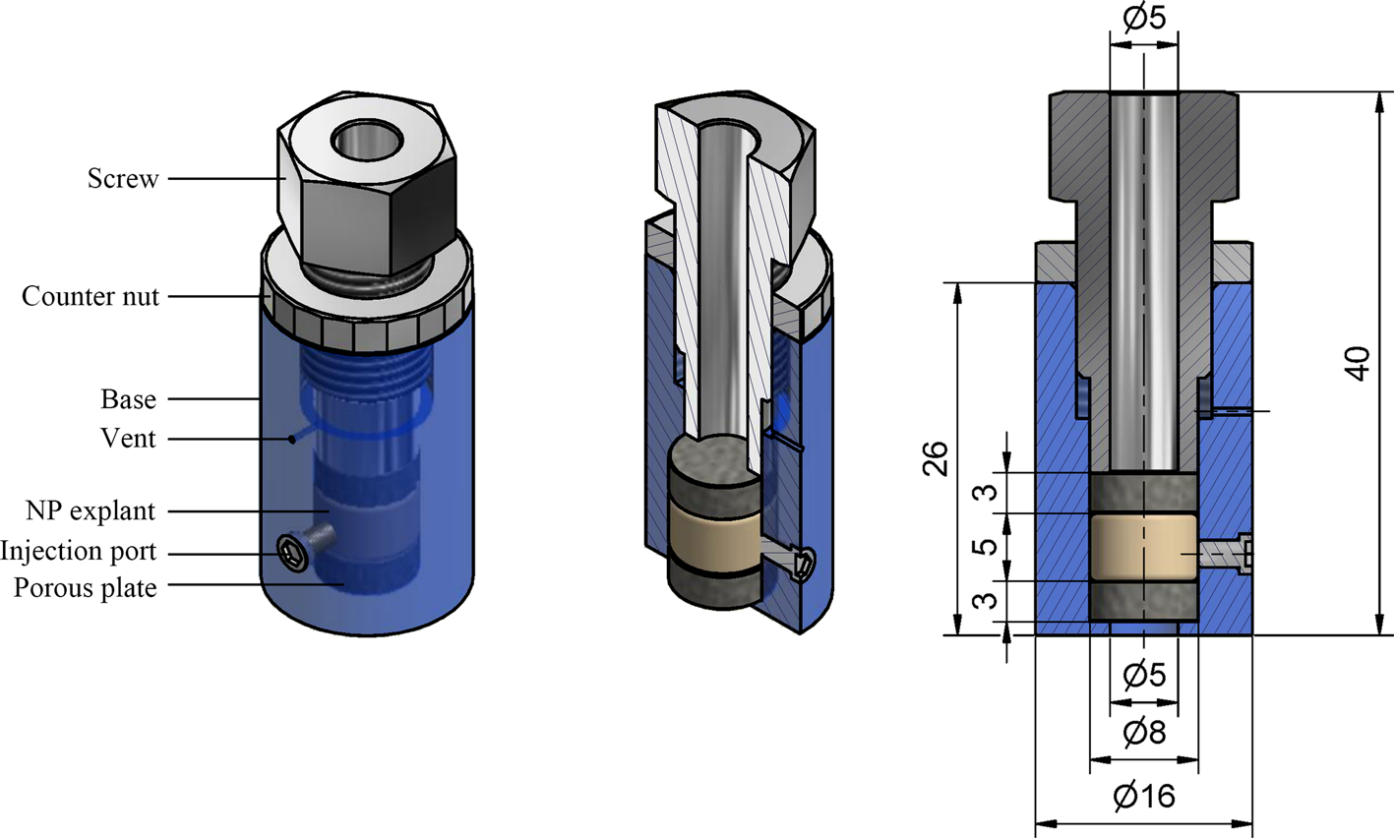
Culture system design.

**Figure 2:**
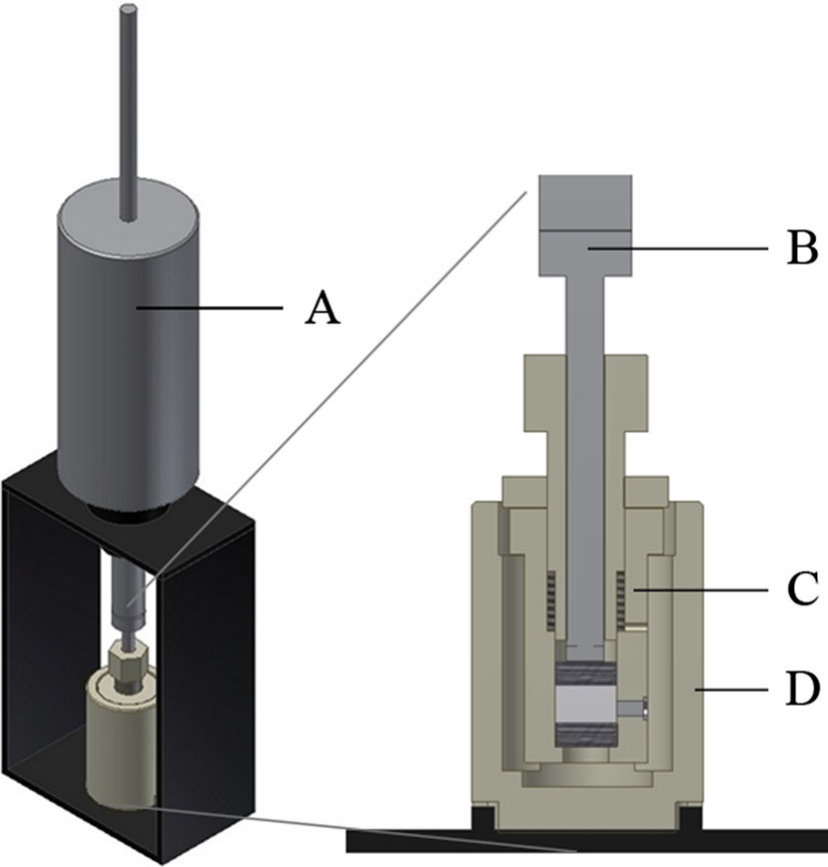
Axial load system design. The axial load system consists of the following components: (a) (replaceable) weight, (b) transmission of load to the upper sintered stainless steel plate, (c) culture system as described in Fig. 1, (d) reservoir for culture medium.

To limit tissue swelling, a system was designed to physically constrain NP tissue in a custom-made hollow cylinder (polyether ether ketone; PEEK) with two porous stainless steel plates (sintered 316L stainless steel; pores 0.5 *μ*m) that allow for molecular transport (Fig. 1). Prior to culture, the position of the upper porous plate, and herewith the tissue’s volume, was set by applying an axial load until equilibrium was reached (Fig. 2). In these experiments, a pressure of 0.3 MPa was applied, and after equilibration (24 h), the position of the upper porous plate was locked. Furthermore, the cylindrical container has an injection port on the side, which can be (re-)closed with a screw.

### Culture System Validation

Fresh bovine tails (24 months old) were obtained from the abattoir according to local regulations. Adjacent to the endplates, the discs (Cy1–Cy5) were opened transversally, and a biopsy punch (ø 8 mm; 273693, Kruuse, Sherburn, UK) was used to harvest tissue from the center of the disc (180–220 mg). NP tissue biopsies were placed in the culture system and equilibrated. After equilibration and subsequent fixation of the tissue volume, the system was placed in a plastic container (60 mL medium) with a lid made of a gas permeable silicone membrane (MED82-5010-10, NuSil, Carpinteria, USA) and cultured at 37 °C, 5% O_2_ and 5% CO_2_. Culture medium consisted of advanced DMEM (12491-015, Invitrogen, Bleiswijk, the Netherlands) supplemented with 10% fetal bovine serum (758093, Greiner Bio-one, Alphen a/d Rijn, the Netherlands) and 1% penicillin/streptomycin (DE17-602E, Lonza, Basel, Switzerland) and was changed twice a week.

To assess long-term culture potential, bovine tails of eight donors were used to obtain eighteen NP samples, which were randomly distributed (disc level and donor) over the following groups:

- *Day 0*, collected immediately after harvesting,
- *Day 1,* collected after equilibration, and
- *Day 28,* collected after 28 days of culture.

For each group, 6 samples (corresponding to 6 donors) were used. The biochemical and water content (¼ sample), histology and cell viability (½ sample) and gene expression (¼ sample) were analyzed.

To visualize injections, bovine tails of three donors were used to obtain six NP samples, which were injected with 10 *µ*L 0.1% Picrosirius red (36.554-8, Sigma, Zwijndrecht, the Netherlands) with a 32 gauge needle (7635-01/00, Hamilton, Bonaduz, Switzerland). Samples were collected 1, 5 or 17 h after injection (*n* = 2/group) to assess diffusion of the dye throughout the tissue.

To assess tissue responsiveness to injected agents bovine tails of nine donors were used to obtain fourteen NP samples, which were randomly distributed (disc level and donor) over the following groups:

- *Sham*, injected with 10 *μ*L of phosphate buffered saline (PBS; *n* = 4),
- *Link*-*N*, injected with 10 *μ*L of 20 *µ*g/*µ*L Link-N peptide (DHLSDNYTLDHDRAIH; CanPeptide Inc; Pointe Claire, QC, Canada; *n* = 5)
- *TNF*-*α*, injected with 10 *μ*L of 0.5 ng/*µ*L recombinant human tumor necrosis factor-α (TNF-α; PHC3016, Invitrogen; *n* = 5),

Injections were administered on day 1, after equilibration, and on day 3. Samples were harvested on day 4. As injections were administered in the center of the samples, the core of each sample was obtained with a 5 mm biopsy punch (Kruuse), and analyzed for gene expression (½ sample).

### Analysis

To assess the biochemical content, samples were weighed (wet weight), lyophilized (Freezone 2.4, Labconco, Kansas City, MO, USA) overnight and then the dry weight was determined. The water content was calculated by dividing the difference between wet and dry weight by the wet weight. Subsequently, the samples were digested overnight in a papain digestion buffer (100 mM phosphate buffer, 5 mM l-cystein, 5 mM ethylenediaminetetraacetic acid and 140 *µ*g/mL papain, all from Sigma) at 60 °C. The DNA content of the digested sample was measured with a Hoechst dye assay^3^ and calf thymus DNA as reference (D3664, Sigma). The glycosaminoglycan (GAG) content was used as a measure for the amount of proteoglycans. The GAG content was determined with a DMMB assay,^5^ using shark cartilage chondroitin sulfate (C4384, Sigma) as reference. The fixed charge density (FCD) was calculated from the GAG per wet weight, as described by Narmoneva *et al.*^19^ The hydroxyproline (HYP) content was measured with a chloramin-T assay^8^ using trans-4-hydroxyproline (H5534, Sigma) as reference.

For histology, samples were snap-frozen in Tissue-Tek O.C.T. compound (4583, Sakura Finetek, Zoeterwoude, the Netherlands) in isopentane in liquid N_2_ and stored at −30 °C until further use. 10 *µ*m thick sections were cut (Microm, Thermo Fisher Scientific, Kalamazoo, MI, USA) to assess cell viability and histology. Lactate dehydrogenase (LDH) staining (N5514, Sigma) to stain living cells dark, as described by Stoddart *et al.*^28^ was used in combination with propidium iodide staining (P3566, Invitrogen) to mark all DNA red-fluorescent to assess the cell viability and distribution. Extracellular matrix was stained with Weigert’s hematoxylin for nuclei, Safranin-O (84120, Fluka, Sigma) for proteoglycans and Fast Green (1.00056.2500, Merck, Darmstadt, Germany) for collagen. Bright field images were made of Safranin-O/Fast Green and LDH sections, while fluorescent images were taken for the LDH staining (Observer, Zeiss, Sliedrecht, the Netherlands).

To measure the gene expression, samples were snap-frozen and stored at −80 °C until further use. Messenger RNA (mRNA) extraction was done as described by van Dijk *et al.*^30^ The concentration of RNA was determined with a spectrophotometer (ND-1000, Isogen, de Meern, the Netherlands). In total, 63 ng of mRNA was used for cDNA synthesis (VILO kit, Invitrogen). Gene expression was analyzed in a real time polymerase chain reaction (qPCR, CFX384, Bio-Rad, Veenendaal, the Netherlands) using the SYBR Green Supermix (Bio-Rad). Three reference genes [*18s* (PrimerDesign, Southampton, UK), glyceraldehyde-3-phosphate dehydrogenase (*GAPDH)*, and *RPL13a*] were evaluated in each experiment, and the most stable one was selected as reference gene. For the long-term culture *18s* was selected and for the short-term tissue response experiment *GAPDH*. The genes of interest were aggrecan (*ACAN*), collagen type I (*COL1*), collagen type II (*COL2*), matrix metalloprotease 13 (*MMP13*), tissue inhibitor of metalloproteinase 1 and 2 (*TIMP1, TIMP2)* for both experiments. Additionally, cyclooxygenase 2 (*COX2*) was tested in the short-term tissue response experiment. Primer sequences are provided in Table 1. Levels of expression were determined using the ΔCt method^13^ and Ct values were normalized to the selected reference gene.

**Table 1:** List of primers for gene expression analysis.

Gene	Accession number	Oligonucleotide sequence (5′ → 3′)
ACAN	NM_173981	F: CCAACGAAACCTATGACGTGTACT
		R: GCACTCGTTGGCTGCCTC
COL1	NM_001034039	F: TGAGAGAGGGGTTGTTGGAC
		R: GGGAGACCATTGAGTCCATC
COL2	NM_001113224	F: TGGCTGACCTGACCTGAC
		R: GGGCGTTTGACTCACTCC
MMP13	NM_174389	F: CCTTGATGCCATAACCAGTCTCC
		R: ATCAATACGGTTGGGAAGTTCTGG
TIMP1	NM_174471	F: GTCAATGAAACTGCCTTATACC
		R: TTCTGGGACCTGTGGAAG
TIMP2	NM_174412	F: GCAACGACATCTACGGCAACC
		R: CCCACACACGGCAGAGGAG
COX2	NM_174445	F: TCCACCAACTTATAATGTGCAC
		R: GGCAGTCATCAGGCACAGGA
GAPDH*	NM_001034034	F: GGCGTGAACCACGAGAAGTATAA
		R: CCCTCCACGATGCCAAAGT
18s*	(Primer Design Ltd)	

* *Reference gene*

ACAN = aggrecan; COL1/COL2 = collagen type I/II; MMP13 = matrix metalloprotease 13; TIMP1/TIMP2 = tissue inhibitor of metalloproteinase 1/2; COX2 = cyclooxygenase 2; GAPDH = glyceraldehydes-3-phosphate dehydrogenase

To visualize picrosirius injections, samples were entirely snap-frozen in Tissue-Tek (Sakura) after culture. 100 *μ*m-thick transversal slices were cut at different heights (top, middle and bottom) of the sample to visualize (Observer, Zeiss) the distribution of the staining inside the sample.

### Statistics

Statistical analysis was performed using R-project software (version 3.0.2).^23^ At first, all data sets were tested for group homogeneity and normal distribution with Levene’s and Shapiro-Wilks’ tests respectively. When homogeneous and normally distributed, a one-way ANOVA was performed with Bonferroni’s corrected *post hoc* independent *t* test. If data were not normal, a Kruskal–Wallis test was done with Bonferroni’s corrected *post hoc* Mann–Whitney test. Statistical significance was assumed for *p* < 0.05.

## Results

### NP Tissue is Maintained in Long-Term Culture

**Figure 3:**
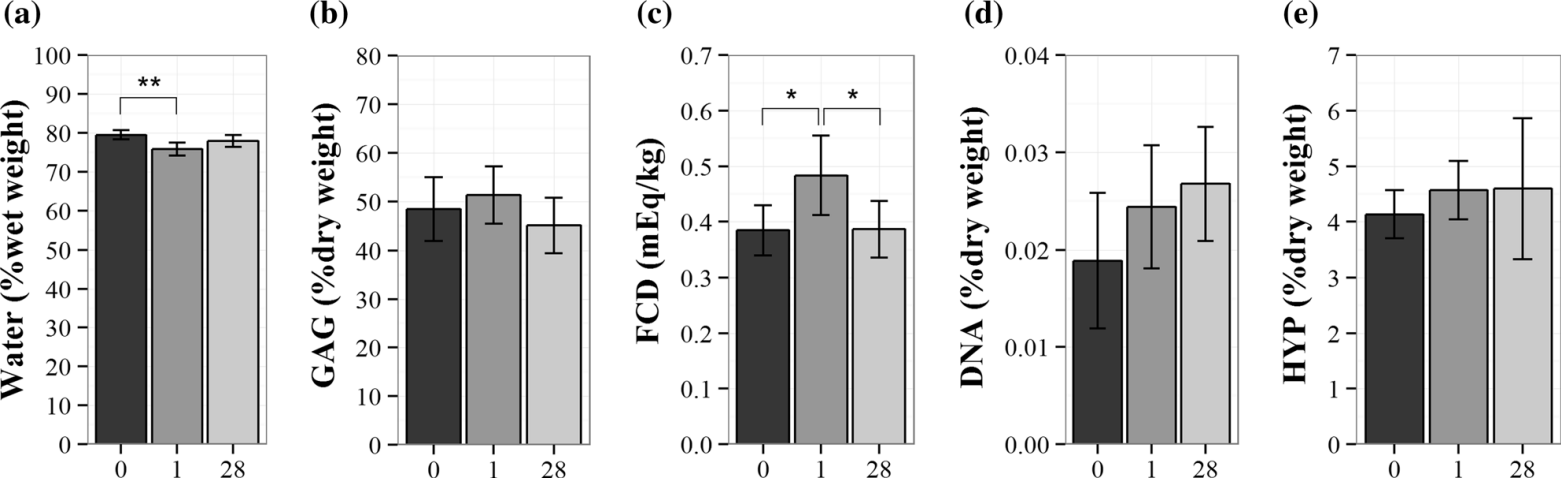
Water and biochemical content after long-term culture. (a) Water content per wet weight, (b) GAG content per dry weight, (c) fixed charged density (FCD), (d) DNA content per dry weight, and (e) hydroxyproline content per dry weight. Values are mean ± SD; *n* = 6 for each group. X-axis = number of days in culture. *p < 0.05 and **p < 0.005.

**Figure 4:**
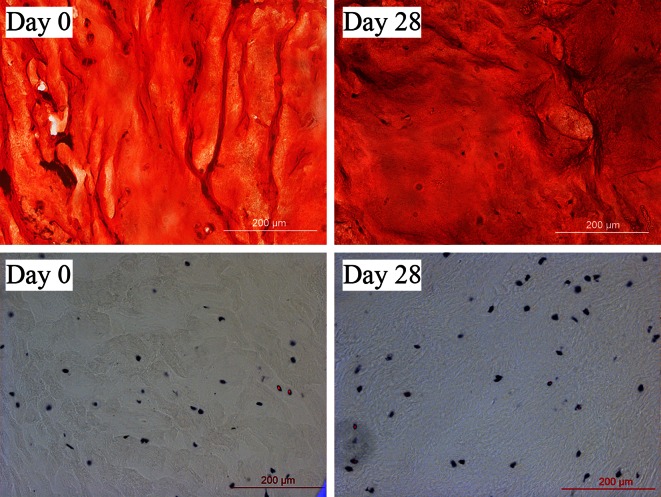
Extracellular matrix and viability staining after long-term culture. Safranin-O/Fast green and LDH staining on day 0 (left) and day 28 (right). Proteoglycans are stained red, collagen green and cell nuclei black in the upper images. Living cells are stained black and their nucleic acids red in the lower images. Scale bars are 200 *μ*m.

**Figure 5:**
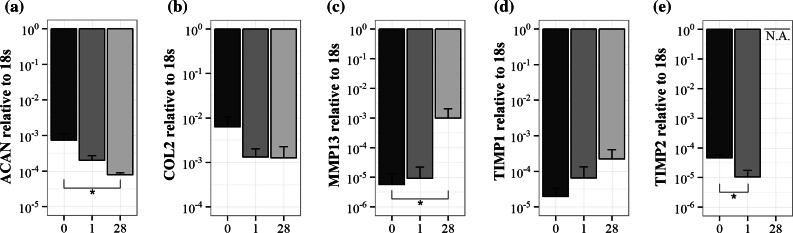
Gene expression after long-term culture. The expression of (a) aggrecan, (b) collagen type II, (c) matrix metalloprotease 13, (d) tissue inhibitor of metalloprotease (TIMP) 1, and (e) TIMP2 relative to reference gene 18s. Please note the logarithmic *y*-axis and error bars. *X*-axis = number of days in culture. Values are mean ± SD; *n* = 5 for day 0 and 1; *n* = 3 for day 28. *p < 0.05.

The water content decreased between day 0 and day 1 (*p* = 0.003), which indicates that water was removed from the tissue during the equilibration phase. By tracking the change in sample height, it could be seen that the equilibrium was reached within 15 h (data not shown). Related to the change in water content, the FCD was higher on day 1 than on day 0 (*p* = 0.02, Fig. 3c). However, by day 28 of culture, the water content and FCD were not significantly different from day 0 (*p* = 0.30 and *p* = 0.95 respectively, Fig. 3a). The total GAG content did not change (*p* = 0.27, Fig. 3b), which was corroborated with the Safranin-O/Fast Green staining (Fig. 4). The collagen content, DNA content (*p* = 0.55 and *p* = 0.15 respectively, Figs. 3d and 3e), and cell viability (Fig. 4) were not different between days of culture. The mRNA expression of *ACAN*, the main extracellular matrix protein, and TIMP2 decreased between day 0 and 28 (*p* = 0.005 and *p* = 0.009 respectively, Figs. 5a and 5e), whereas *MMP13* expression increased in the same time frame (*p* = 0.03, Fig. 5c). *COL2* and *TIMP1* expression did not change significantly (*p* = 0.08 and *p* = 0.21 respectively) during the culture period (Figs. 5b and 5d), while the expression of *COL1* was beyond detection limits for all samples.

### Injections can be Targeted

**Figure 6:**
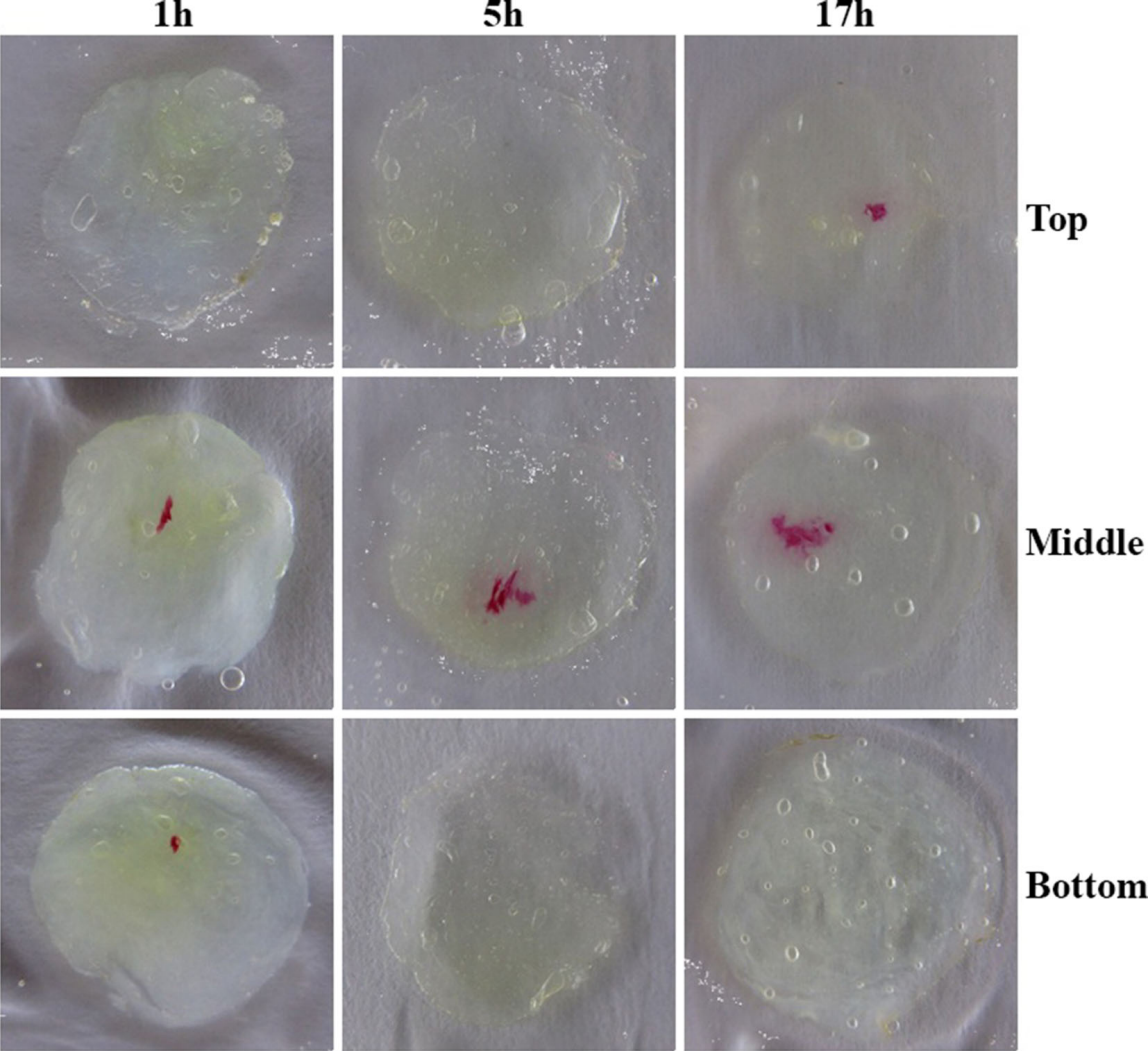
Targeting injections. Diffusion was observed 1, 5 or 17 h after injection of picrosirius red in the top, middle or bottom part of the sample.

Picrosirius red staining showed that targeting an injection in the core of the NP tissue is feasible. Time dependent picrosirius diffusion was observed, as the intensity of the color decreased in the core and increased in the surrounding areas (Fig. 6).

### NP Tissue is Responsive to TNF-α

**Figure 7:**
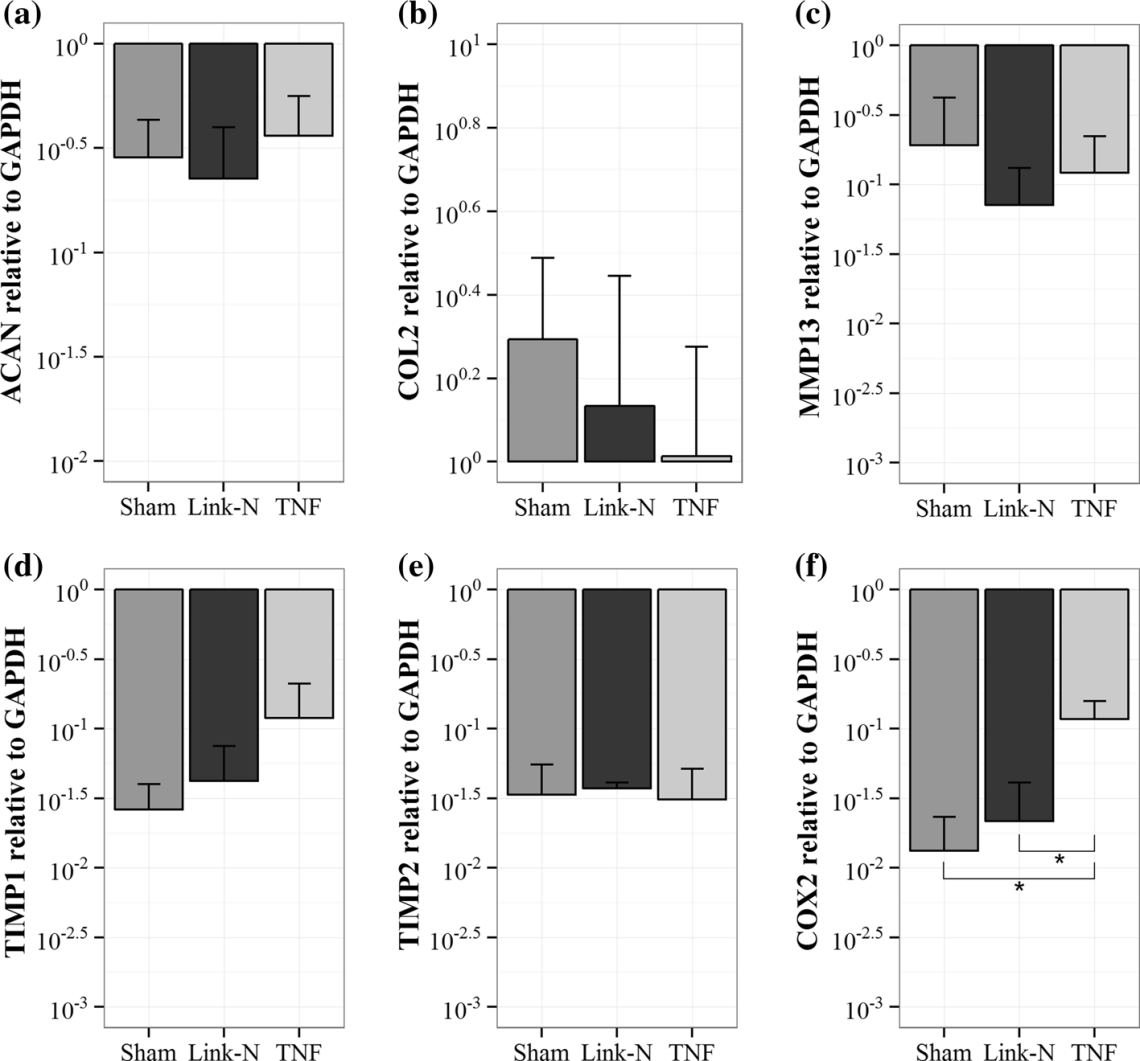
Gene expression after injections of Link-N and TNF-α. The expression of (a) aggrecan, (b) collagen type II, (c) matrix metalloprotease 13, (d) tissue inhibitor of metalloprotease (TIMP) 1, (e) TIMP2, and (f) cyclooxygenase 2 relative to reference gene glyceraldehyde-3-phosphate dehydrogenase. Please note the logarithmic *y*-axis and error bars. *X*-axis = injection group. Values are mean ± SD; *n* = 3 for sham; *n* = 4 for Link-N; *n* = 5 for TNF-α. *p < 0.05.

*COX2* expression was significantly higher in the samples injected with TNF-α than in the sham and Link-N groups (*p* = 0.02 and *p* = 0.04 respectively, Fig. 7f), which shows that NP tissue is responsive in this system. Injections of Link-N did not result in a change in gene expression when compared to the sham group (all genes, *p* > 0.40; Figs. 7a–e). The expression of *COL1* was beyond detection limits for all samples.

## Discussion

It was previously shown that constraining NP tissue maintains the extracellular matrix and mostly preserves native gene expression profiles in long-term culture.^30^ However, constraining of the tissue was done manually and reproducibility was less than desired. Furthermore, the actual osmolarity and hydraulic pressure in the NP were unknown.^30^ By controlling the initial osmotic pressure, a novel and very reproducible NP culture system was designed where a fixed tissue volume could be created as a function of the initial osmotic pressure.

Previous studies have shown adverse effects on gene expression and matrix composition if static compression of 0.4 MPa or higher was applied.^14^,^32^ Therefore, the axial load for equilibration in this study was chosen to reach an osmotic pressure of approximately 0.3 MPa, as the osmotic pressure range at rest in humans is 0.1–0.3 MPa *in vitro*.^33^ Equilibration with 0.3 MPa caused some water loss initially, resulting in an increased FCD and osmotic pressure at day 1. Nevertheless, after 28 days of culture the water content and FCD returned to that in freshly harvested tissue. While no statistically significant changes in the GAG content and histology were observed compared to day 1, it is possible that a small (quantitatively insignificant) amount of GAG was lost. As aggrecan is partly fragmented, even in healthy IVD tissue,^26^ fragments could have diffused out of the tissue between day 0 and 28. This GAG loss could have caused a decrease in FCD and osmotic pressure. Without a change in hydraulic pressure this would have resulted in a loss of water, but with a reduction of the tissue’s volume accompanying the loss of GAGs, the drop in hydraulic pressure could have been equal or greater than the drop in osmotic pressure. This caused either no exchange of water or a slight influx into the tissue until osmotic and hydraulic pressure were again equal. Therefore, in hindsight 0.3 MPa may have been a suboptimal equilibrium load, as self-regulation was necessary to maintain the tissue. Nevertheless, the extracellular matrix composition and cell viability remained statistically unchanged in long-term culture, thus the decrease in osmotic pressure was small and changes in tissue content were statistically insignificant.

However, the expression of some genes did change. The expression of main matrix component *ACAN* decreased, like in some previous long-term NP cultures.^30^ Also in this respect, the 0.3 MPa during equilibration may have been suboptimal, and an optimal pressure, at which both extracellular matrix and gene expression are maintained, may exist and should be explored. However, additional influential factors may also be considered. Previously, it has been shown that anabolic gene expression of isolated NP cells (NPCs) in hydrogels increases after dynamic hydrostatic loading,^15^,^20^ that mildly degenerated rat NPs show increased *ACAN* expression after dynamic axial compression,^34^ and that unloaded caprine discs show a significantly lower *ACAN* expression in their NPs than the NPs of loaded discs,^22^ if the applied forces are within the physiological range. Since both dynamic hydrostatic loading and dynamic compression can be applied on the NP in this culture system, by transmission through or displacement of the sintered stainless steel plates, loading regimens can be used to mimic the physiological conditions and herewith possibly maintain *ACAN* gene expression at native levels. Finally, gene expression profiles may be improved by lowering the pH to 7.1–7.2, as it has been shown that at this physiological pH the proteoglycan synthesis rate is highest *in vitro*,^21^ and by culturing in serum-free medium.^24^ Based on previous studies^7^,^16^,^30^ the oxygen concentration was chosen at 5% to create a physiological gradient towards the center of the NP.^18^

Needles with gauges of a wide range can be used to inject into the tissue through the injection port. Therefore, it is possible to inject controlled release hydrogels, large cells (e.g., notochordal cells) and therapeutics. In the current study sample heights were very similar, so that the deposition of the injected material was easily centralized in the core of the samples. In case that sample heights differ, some more care regarding the needle depth and skewedness is required to inject in the core.

In this study, PBS, Link-N and TNF-α were injected in bovine NP tissue to test if the tissue is able to respond in the culture system. Synthetic peptide Link-N has been shown to be a promising therapeutic agent for NP regeneration, as it increases proteoglycan synthesis,^17^*ACAN* expression, and simultaneously decreases the expression of catabolic markers in isolated NPCs.^6^ Similarly, Link-N was able to increase proteoglycan synthesis *in vitro* in intact human IVDs.^6^ However, in the current study no increase in *ACAN* expression was observed after two Link-N injections. The discrepancy between the results of the current study and that of Gawri *et al.* may be explained by the culture method. Link-N molecules are very small (1.9 kDa) and therefore they can freely diffuse through the tissue. Unlike the study of Gawri *et al.* where Link-N, injected into the NP of a whole disc, is kept inside the disc by the rather impermeable AF and endplates, our system was not able to prevent Link-N diffusion out of the system. Furthermore, in the current study, the injected amount of Link-N was not corrected for the (very large) volume of culture medium, in which Link-N diffused. Picrosirius red, used for testing the injection port, has a similar molecular weight as Link-N and diffused throughout the whole tissue within 17 h. In this respect, it is likely that the final tissue concentration of Link-N in the current study was below the effectiveness level. For future studies, it is recommended to inject a controlled release hydrogel containing Link-N, in order to keep Link-N available to the cells during the full period of culture. Alternatively, a larger growth factor like osteogenic protein-1 (OP-1; a.k.a. BMP-7) may be used. Due to their size, the diffusion of these molecules is expected to be slower.

TNF-α (17.5 kDa) is one of the major inflammatory cytokines measured during herniation and degeneration of the NP.^25^,^35^ In this study, TNF-α injections resulted in an increased *COX2* expression compared to the sham and Link-N groups. Up regulation of *COX2* expression in response to TNF-α stimulation is in accordance with literature that describes the effect of TNF-α on isolated NPCs^29^ or in tissue engineered NP.^27^ In addition to *COX2* up regulation, the latter study showed down regulation of *ACAN* expression after TNF-α supplementation, which was not observed in the current study.^27^ This discrepancy could be explained by the difference in experimental set-up between the studies: cells isolated from their native environment may not fully maintain their phenotype during the isolation procedure and/or they may behave differently when no native extracellular matrix is present.^9^ The differences between the studies emphasize the added value of culturing NP tissue compared to isolated NPC culture only. In these culture systems the NP tissue can be maintained and responsive under physiological conditions. IVD organ culture systems additionally include interactions with the other tissues of the IVD, but maintaining the tissue for longer periods is difficult.^4^,^10^ The *in vivo* testing of therapeutics is most attractive, but expensive and not ethical in early stages of research.^1^

In conclusion, the native biochemical content and cell viability of NP tissue were maintained when cultured at a fixed tissue volume after equilibration with an osmotic pressure of 0.3 MPa for 28 days. In addition, injections of cytokine TNF-α showed that the tissue is responsive. This novel culture system can be used to culture NP explants in a very reproducible manner and to test potential regenerative therapies.
